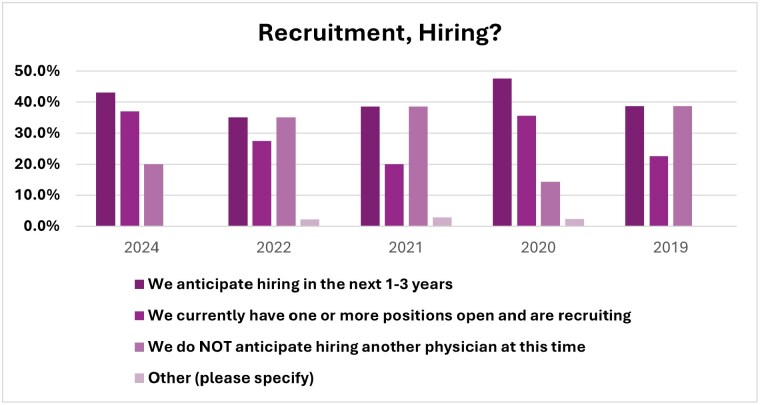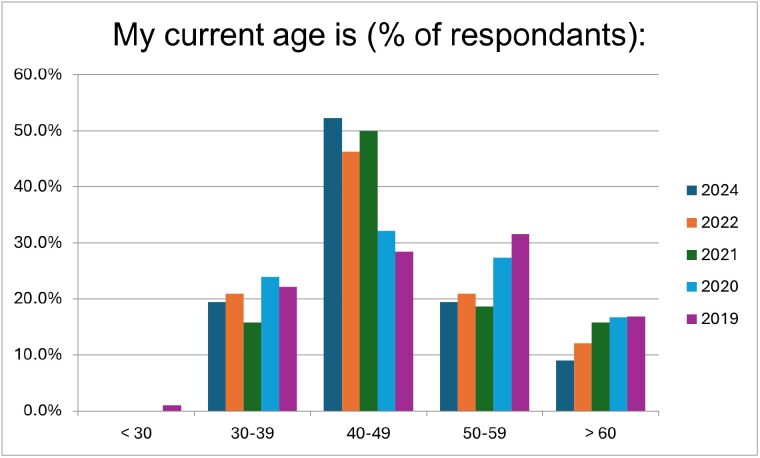# 615 Burn Surgeons - A Recap of Highlighted Workforce Data 2018-2024

**DOI:** 10.1093/jbcr/iraf019.244

**Published:** 2025-04-01

**Authors:** Randy Kearns, Jeffrey Carter, Kathleen Romanowski

**Affiliations:** University of New Orleans; Louisiana State University Burn Center; University of California, Davis and Shriners Hospitals for Children

## Abstract

**Introduction:**

Since 2018, the Organization for the Delivery of Burn Care (ODBC) workforce subcommittee for the American Burn Association (ABA) has conducted a yearly survey of burn surgeons (ABA members). This survey is designed to capture critical traits and longitudinally identify emerging trends related to burn surgeons.

**Methods:**

The workforce survey of burn surgeons has included demographics and other emerging trends for members of the past five years.

2024 Survey

Survey distributed Feb 2024

Closed Mar 2024

Results tallied Jun 2024

Research Design = Descriptive Analysis with 5-year longitudinal results where available

**Results:**

The ABA collected and anonymized the results before sending them to the ODBC workforce subcommittee co-chair (non-surgeon business school professor). The data were analyzed and aggregated there before being reported to the burn surgeon community. The number of burn surgeons completing at least a portion of the survey totaled 81 for 2024. Historically, this compares to 113 for 2023, 91 in 2021, 98 in 2020, and 139 in 2019.

Age

For the past three years, the largest age group has been 40-49, comprising over 50% of the respondents in 2024. The over >60 age group is the only one to see a steady decline: 16.8% (2019), 16.7% (2020), 15.7% (2021), 12.1% (2022), and 9.0% (2024).

Gender

While this remains a “Man/Male” dominated profession, the 2020 M/F mix (67.9%/31.0%) compared to the 2024 M/F mix (59.7%/38.8%) reflects the growth of Female burn surgeons.

Other Roles

Focusing on how much of the role is committed to providing care for a patient with a burn injury, respondents have consistently reported (>60% of all respondents) that their clinical role is caring for patients with burn injuries (≥80% of the time).

Practice Location and Academic Rank

While burn care continues to be provided in community hospitals (13.4%), most burn surgeons report working in a university/medical center setting and hold a university rank (86.6%).

Growth Trends Two questions focus on the future of physician recruitment and the need for additional burn surgeons. One question targeted local practice groups’ health and growth trends, which indicates growth (47.8% [2024] vs. 40.7% [2022]) was the leading response for the first time in the survey’s history.

The second question included the current state of recruiting. Overwhelmingly, (65%) are either currently recruiting or anticipate hiring in the next 1-3 years.

**Conclusions:**

This report is a selected review of the workforce data, focusing on demographics and several highlights from the annual workforce survey. The industry is experiencing healthy trends, including growth in the need for burn surgeons and a cohort of younger surgeons filling the shoes of the retiring (or retired) pioneers who first defined the profession.

**Applicability of Research to Practice:**

There are concerns about the availability of the burn surgeon workforce, but there are some positive trends.

**Funding for the Study:**

N/A